# Unraveling the Antioxidant, Antihyperlipidemic, and Antidiabetic Potential of *Jatropha integerrima* in Streptozotocin-Induced Diabetic Rats

**DOI:** 10.3390/life16020246

**Published:** 2026-02-02

**Authors:** Deepak Bharati, Dixitkumar Pualsa, Shreya Mayekar, Jegan Nadar, Popat Mohite, Ashwini Kumar, Sudarshan Singh

**Affiliations:** 1 Department of Pharmacology, AETs St. John Institute of Pharmacy and Research, Palghar 401404, Maharashtra, India; deepakb@sjipr.edu.in (D.B.); pualsa.dixit@gmail.com (D.P.); shreyamayekar00@gmail.com (S.M.); jegann@sjipr.edu.in (J.N.); 2 Department of Chemistry, AETs St. John Institute of Pharmacy and Research, Palghar 401404, Maharashtra, India; 3 Research and Development Cell, Manav Rachna International Institute of Research and Studies, Faridabad 121003, Haryana, India; aknitjsr08@gmail.com; 4 Faculty of Pharmacy, Chiang Mai University, Chiang Mai 50200, Thailand; 5 Office of Research Administration, Chiang Mai University, Chiang Mai 50200, Thailand

**Keywords:** antidiabetic, antioxidant, *Jatropa integerrima*, antihyperlipidemic, streptozotocin

## Abstract

Diabetes *mellitus* (DM) is a chronic metabolic disorder associated with hyperglycemia, oxidative stress, and dyslipidemia, leading to severe complications. Medicinal plants like *Jatropha integerrima*, known for their antioxidant and therapeutic properties, are being explored as potential alternatives for the management of diabetes. The present study aimed to evaluate the antidiabetic, antihyperlipidemic, and antioxidant effects of the methanolic extract of *Jatropha integerrima* (MEJI) in streptozotocin (STZ)-induced diabetic rats. Diabetes was induced in Wistar rats using STZ (45 mg/kg, i.p.), followed by oral treatment with MEJI (200 and 400 mg/kg) or metformin (200 mg/kg) for 21 days. Glycemic control was assessed through fasting blood glucose level (FBG), and the oral glucose tolerance test (OGTT), lipid profiling (TC, TG, LDL, HDL, and VLDL), and antioxidant (SOD and CAT) testing were outsourced to UNIQUE Biodiagnostics Vet. Path Lab, Parel, Maharashtra, while pancreatic histopathology was analyzed by evaluating islet morphology. Treatment with MEJI produced a dose-dependent reduction in fasting blood glucose levels. On day 21, MEJI at 200 and 400 mg/kg reduced blood glucose by 63.1% and 67.0%, respectively, compared to the diabetic control group. The standard drug showed the highest reduction (73.6%), restoring glucose levels close to normal values, compared with the diabetic control group, along with an improvement in glucose tolerance as reflected in OGTT outcomes. Moreover, the extract also favorably modulated the lipid profile by lowering TC, TG, LDL, and VLDL levels while enhancing HDL concentrations. Antioxidant enzyme activities improved notably, with significant elevations in SOD and CAT levels, indicating attenuation of oxidative stress. Furthermore, the histopathological examination of pancreatic sections revealed partial recovery of islet architecture in MEJI-treated rats, suggesting regenerative and protective effects on pancreatic β-cells. MEJI exhibited potent glucose-lowering, lipid-regulating, and antioxidant properties, along with pancreatic protection. These findings suggest that *Jatropha integerrima* may serve as a reservoir of bioactive compounds with promising potential for the management of diabetes.

## 1. Introduction

Diabetes *mellitus* (DM) is a chronic, progressive, and multifunctional metabolic disorder marked by sustained hyperglycemia due to a combination of insulin resistance and relative insulin deficiency [1]. Representing approximately 90–95% of all diabetes cases worldwide, type 2 diabetes *mellitus* (T2DM) is strongly associated with a sedentary lifestyle, obesity, poor dietary habits, and genetic predisposition [2,3]. According to the International Diabetes Federation, an estimated 537 million adults globally were living with diabetes in 2021, with the prevalence projected to reach 643 million by 2030 and 783 million by 2045 [4]. This alarming rise underscores the urgent need for effective, safe, and affordable therapeutic strategies [5]. The pathophysiology of T2DM involves impaired insulin signaling, excessive hepatic glucose production, dysregulated lipid metabolism, and chronic oxidative stress. Persistent hyperglycemia leads to the overproduction of reactive oxygen species (ROS), which surpass the capacity of the body’s endogenous antioxidant systems, resulting in oxidative damage to protein, lipids, and DNA [6,7]. Furthermore, diabetic dyslipidemia is characterized by elevated total cholesterol (TC), triglycerides (TG), low-density lipoprotein cholesterol (LDL-C), and reduced high-density lipoprotein cholesterol (HDL-C) that significantly contribute to the development of cardiovascular complications in T2DM patients [8].

Current pharmacological therapies, including metformin, sulfonylureas, and DPP-4 inhibitors, have shown efficacy in managing blood glucose levels [9]. However, their long-term use is often associated with limitations such as gastrointestinal side effects, weight gain, hypoglycemia, and inadequate control of oxidative stress and lipid abnormalities [10,11]. Plant-derived therapeutics are exploratory candidates requiring rigorous pharmacological and toxicological validation, rather than proven solutions [12]. Medicinal plants are a rich source of bioactive phytoconstituents, such as flavonoids, phenolics, tannins, terpenoids, and alkaloids, which exert antidiabetic effects through various mechanisms, including enhancement of insulin secretion, improvement in insulin sensitivity, inhibition of carbohydrate-digesting enzymes, modulation of glucose uptake, and antioxidant activities [13,14].

*Jatropha integerrima* Jacq., a member of the Euphorbiaceae family, is an evergreen ornamental shrub widely distributed in tropical and subtropical regions that originates from Cuba and Hispaniola (the West Indies). Traditionally, it has been used to treat inflammatory conditions, microbial infections, and skin disorders [15,16]. It also shows antioxidant, anti-inflammatory, and anti-neurodegenerative activities and cytotoxic potential [17,18]. Phytochemical screenings of *J. integerrima* have confirmed the presence of flavonoids, phenolic compounds, alkaloids, glycosides, and saponin compounds known for their antidiabetic and antioxidant properties [19]. Despite its promising ethnomedicinal profile, scientific validation of its therapeutic efficacy in diabetes management remains limited and not reported earlier. The present study was undertaken to investigate the antidiabetic, antioxidant, and antihyperlipidemic effects of the methanolic extract of *Jatropha integerrima* (MEJI) in a streptozotocin (STZ)-induced diabetic rat model. STZ at sub-diabetogenic doses, often in combination with dietary modifications, can mimic late-stage T2DM by inducing partial pancreatic β-cell dysfunction [20]. This study evaluated MEJI’s effects on fasting blood glucose, oral glucose tolerance, lipid profile, antioxidant enzyme activities, superoxide dismutase (SOD) and catalase (CAT), and pancreatic histopathology. Metformin was used as a standard reference drug. Moreover, this study aims to bridge the gap between traditional knowledge and scientific validation by exploring the therapeutic promise of *Jatropha integerrima* as a potential agent for integrated T2DM management.

## 2. Materials and Methods

### 2.1. Chemicals and Reagents

The standard drug, metformin, was obtained from Aarti Drugs Limited, Boisar, India. The standard drugs used for gallic acid and quercetin were procured from Yucca Enterprises, Mumbai, India. Methanol, ammonia, hydrochloric acid, acetic anhydride, formic acid, ethyl acetate, sodium citrate, citric acid, ascorbic acid, aluminum chloride, sodium hydroxide, sulfuric acid, glacial acetic acid, and petroleum ether were obtained fromSigma-Aldrich, Mumbai, India, and streptozotocin (STZ) was obtained from Sisco Research Laboratories, Andheri, India. All the chemicals and reagents were of analytical grade.

### 2.2. Collection and Authentication of Plant Material

Fresh leaves of *Jatropha integerrima* were collected from the campus of St. John Institute of Pharmacy and Research, Palghar, Maharashtra, during November 2024. The collected leaves were thoroughly washed with distilled water, shade-dried at room temperature for 7–10 days, and coarsely powdered using a mechanical grinder. The powdered material was stored in an airtight container under dry conditions, and further taxonomical and morphological traits were identified by Dr. R. K. Choudhary, scientist at the Agharkar Research Institute, Pune, India, under the authentication number AUTH 24-213.

### 2.3. Preparation of Methanolic Extraction of J. integerrima

The leaves of *J. integerrima* were freshly collected from healthy trees and meticulously washed. After washing, the leaves were dried in an oven at 45 °C to obliterate any remaining moisture content. Once thoroughly dried, the leaves were finely powdered and subjected to an extraction method using a Soxhlet apparatus. First, the powdered leaves were defatted using petroleum ether as the solvent for 72 h, which allowed for the removal of any fatty substances. This was further followed by a maceration with methanol for an additional 48 h to isolate the non-polar compounds present in the leaves. The resulting solvent mixture was then carefully filtered using Whatman filter paper no. 1 to remove the solid plant material, ensuring that only the liquid extract was retained. The solvent was then evaporated using an electric water bath at 45 °C to concentrate the extracts into a more potent form. The percentage yield of the extract was calculated and stored in an airtight container at 4 °C.

### 2.4. Qualitative Phytochemical Analysis

The extracts were screened for the presence of secondary metabolites such as alkaloids, saponins, flavonoids, phenolic hydroquinones, triterpenoids, and tannins, as reported previously [21]. All solvents used were analytical grade, and phytochemical contents were detected qualitatively. Qualitative phytochemical screening of the extract was carried out using standard chemical tests to identify the presence of major classes of secondary metabolites. Alkaloids were detected using Dragendorff’s, Mayer’s, and Wagner’s reagents. The presence of flavonoids was evaluated by the Shinoda test and the alkaline reagent test. Phenolic compounds and tannins were identified using the ferric chloride test and the lead acetate test. Saponins were assessed by the froth formation test, while terpenoids were detected using the Salkowski test. The presence of steroids was confirmed by the Liebermann–Burchard reaction. Glycosides were screened using the Keller–Killiani test, and carbohydrates were identified by Molisch’s test. These qualitative analyses provided an overview of the phytochemical composition of the extract and supported the interpretation of its observed biological activity.

### 2.5. Quantitative Analysis

#### 2.5.1. Total Flavonoid Content

The flavonoid content within the *J. integerrima* extract was tested as reported in [22]. In brief, the aluminum chloride colorimetric technique was utilized to estimate the MEJI total flavonoid content. For the calibration curve, quercetin had been used as the reference standard. Working standards were prepared by serial diluting to concentrations of 10, 20, 30, 40, 50, and 60 ppm in methanol after 5 mg of quercetin was dissolved in 1.0 mL of methanol to produce a stock solution. A total of 0.6 mL of a 2% aluminum chloride solution was used to dissolve 0.6 mL of each quercetin standard or plant extract for the test. Later, for 60 min, the mixtures were allowed to stand at room temperature. A UV-Visible spectrophotometer (Shimadzu UV VIS Spectrometer 1900, Kyoto, Japan) was then used to measure the absorbance at 420 nm in comparison to a blank. The flavonoid content in the samples has been assessed using the calibration curve (y = 0.0111x + 0.0757) and was clearly expressed as quercetin equivalents (mg/g of extract) and calculated using formula TFC = (C × V)/M.

#### 2.5.2. Total Phenol Content

The total phenolic content within the *J. integerrima* extract was tested as reported in [23]. Briefly, stock solution of gallic acid (10 mg/100 mL distilled water) was prepared, sonicated (15 min), and filtered. Working standards (20–100 ppm) were obtained by appropriate dilutions. MEJI solution (100 mg/10 mL water) was similarly prepared, filtered, and diluted to 40 ppm. For the determination of total phenolic content, the sample was first mixed with 0.5 mL of 2N Folin–Ciocalteu reagent; after an incubation period of 4 min, 1.5 mL of 0.7 M sodium carbonate was added, and the reaction mixture was finally diluted to the required volume with distilled water. The mixture was vortexed and incubated in the dark for 40 min, and absorbance was measured at 725 nm using Shimadzu UV-VIS Spectrometer 1900, Japan. Total phenolic content was clearly expressed as gallic acid equivalents (mg/g of extract) using a calibration curve (y = 0.0059x + 0.0887). All assays were performed in triplicate.

Total phenolic content (TPC) was calculated using the following formula:

TPC=C×V/W

where C is the concentration of phenolic compounds determined from the gallic acid calibration curve (mg/mL), V is the volume of extract used in the assay (mL), and W is the weight of the extract (g). The results were expressed as mg of gallic acid equivalents (GAEs) per g of extract.

### 2.6. In Vitro Radical Scavenging Assay

The in vitro antioxidant potential of *J. integerrima* extract was tested as reported in [24]. Briefly, a 10 mg DPPH in 100 mL methanol was kept for 30 min in the dark for the reaction. For sample and standard preparation, mix 50 mg of the methanolic extract of *J. integerrima* (MEJI) and 50 mg of ascorbic acid with 100 mL of methanol, respectively. Sonicate for 15 min and filter. Prepare 5 concentrations (10, 20, 30, 40, and 50 ppm) by diluting 1 mL of each concentration with 3 mL of DPPH stock solution and adding methanol until reaching 10 mL. The absorbance was measured at 517 nm using a UV-Visible spectrophotometer (Shimadzu UV-Vis 1900), and IC50 was calculated. The experiment was performed in triplicate to determine the radical scavenging activity of sample tested.

### 2.7. In Vivo Study

#### 2.7.1. Ethical Approval

All experimental protocols involving animals were reviewed and approved by the Institutional Animal Ethics Committee (IAEC) of St. John Institute of Pharmacy and Research, India (IAEC Reference No: SJIPR/IAEC/05/24-25 dated 12 August 2024). This study was conducted in accordance with the guidelines of the Committee for the Control and Supervision of Experiments on Animals (CCSEA), Government of India.

#### 2.7.2. Experimental Animals

A total of 30 male albino Wistar rats (200–250 g) were procured from Lascmi Biopharms Pvt Ltd., Pune, India [Reg.No.1277/CCSEA]. The animals were acclimatized for ten days under standard housing conditions in an animal house approved by the Committee for Control and Supervision of Experiments on Animals (CCSEA). Animals were housed in polypropylene cages and maintained at a temperature of 22 ± 3 °C, with a relative humidity of 50 ± 10%, under a 12 h light/dark cycle. Moreover, the animals were allowed free access to a standard laboratory diet and water ad libitum.

#### 2.7.3. Acute Oral Toxicity

Acute oral toxicity study of the methanolic extract of *J. integerrima* was performed according to the Organization of Economic Co-operation and Development (OECD) Guideline No. 425 (Up-and-Down Procedure) [25].

#### 2.7.4. Induction of Diabetes in Wistar Rats

The diabetes was induced by a single intraperitoneal injection of streptozotocin as reported in [26]. Briefly, experimental diabetes was induced in overnight-fasted Wistar albino rats by a single intraperitoneal injection of streptozotocin (STZ; 45 mg/kg of body weight) freshly dissolved in ice-cold citrate buffer (0.1 M, pH 4.5). To prevent initial hypoglycemia, animals were supplied with 10% glucose solution ad libitum for 24 h post-injection. Seventy-two hours later, fasting blood glucose (FBG) was measured using a glucometer (BG-03 Dr. Morepen Glucometer, Morepen Laboratories Limited, Baddi, India), and rats with FBG ≥ 250 mg/dL were considered diabetic and included in this study.

#### 2.7.5. Experimental Design

No mortality was observed at the acute oral dose of 5000 mg/kg of body weight after oral route. Therefore, the median lethal dose following oral administration was 5000 mg/kg of body weight. For the selection of test doses, 12.5% and 25% of 5000 mg/kg of body weight were used as concentrations of doses for *J. integerrima* extract. Five normal healthy rats were chosen randomly for the control group. Twenty diabetic-induced rats were selected, and five rats were randomly assigned to each group for this study.

Group I: Control rats orally administered with distilled water.
Group II: STZ-induced diabetic rats administered orally with distilled water.
Group III: STZ-induced diabetic rats administered orally with metformin (200 mg/kg) dissolved in distilled water.
Group IV: STZ-induced diabetic rats administered orally with the methanolic extract of *J. integerrima* (200 mg/kg).
Group V: STZ-induced diabetic rats administered orally with the methanolic extract of *J. integerrima* (400 mg/kg). All the treatments were started on the fourth day after STZ injection and once a day, which continued for twenty-one days. Fasting blood glucose and body weight were recorded on days 0, 4, 7, 14, and 21.

#### 2.7.6. Oral Glucose Tolerance Test

On day 22, all rats were fasted overnight before the oral glucose tolerance test (OGTT). The fasting blood glucose (FBG) levels were recorded, after which the animals received their respective oral treatments: normal control and diabetic control groups received water, the standard control group received metformin (200 mg/kg, p.o.), while the treatment groups received the methanolic extract of *Jatropha integerrima* at doses of 200 mg/kg and 400 mg/kg, respectively. Thirty minutes after the treatments, each rat was administered an oral glucose load of 2 g/kg of body weight via oral gavage [27]. Blood glucose levels were measured from the tail vein at 0 min (just before glucose administration) and at 30, 60, 90, and 120 min post-glucose administration using a glucometer (Dr. Morepen Gluco One BG-03 Glucometer, Morpen Laboratories Limited, Baddi, India). The area under the curve (AUC) for blood glucose concentration over time was calculated to assess glucose tolerance. Briefly, the AUC was determined from the glucose time profile using the trapezoidal rule, in which the area between successive time points was calculated as the average of the two consecutive blood glucose values multiplied by the time interval between them. The individual trapezoid areas obtained the total AUC for each animal. The resulting AUC values reflect the overall glycemic exposure during the OGTT, with lower AUC indicating improved glucose tolerance.

### 2.8. Biochemical Analysis

The biochemical analysis was tested as reported in [28]. In brief, on days 0, 4, 7, 14, and 21 of treatment, blood samples were collected from the retro-orbital plexus. The blood was centrifuged at 3000 rpm for 5 min. Using an automated chemistry analyzer, the supernatant was then separated from the precipitate, and serum samples were prepared to determine the levels of TC, TG, HDL, and LDL.

### 2.9. Estimation of SOD and CAT

SOD and CAT activities were evaluated as previously reported [29,30]. Antioxidant effect was evaluated through the modulation of endogenous antioxidant enzymes (SOD and CAT) in the tissue homogenate of isolated liver. In brief, on day 22, one animal from each group was sacrificed, and pancreatic tissue was collected and preserved in saline for analysis of oxidative stress parameters. SOD activity was determined based on inhibition of pyrogallol auto-oxidation at 420 nm and expressed as units/mg protein. CAT activity was assayed by monitoring the rate of decomposition of hydrogen peroxide (H_2_O_2_) at 240 nm and expressed as units/mg of protein. Evaluation of oxidative stress markers was outsourced to UNIQUE Biodiagnostics Vet. Path Lab, Parel, Maharashtra, India.

### 2.10. Histopathological Study

The pancreas from each group of animals was quickly removed after sacrificing the animals and washed immediately in ice-cold saline. Each small portion of tissue was fixed in 10% neutral formalin fixative solution for carrying out histological studies. Tissue was embedded in paraffin after fixation, and then solid sections were cut at 5 μm, which were then stained with hematoxylin and eosin. The photomicrographs of pancreatic sections were captured using a digital camera attached to the light microscope.

### 2.11. Molecular Docking

The molecular docking analysis was conducted using PyRx software (version 0.8). The binding sites were defined based on the co-crystallized ligands from the protein crystal structures obtained from the Protein Data Bank (https://www.rcsb.org/), accessed on 10 December 2025 (PDB codes: 1UOK and 3DH4). To prepare the proteins for docking, water molecules and non-essential heteroatoms were removed. The protein structures were further cleaned by adding missing hydrogen atoms and optimizing rotatable bonds where applicable. The active sites were identified according to the coordinates of the original ligands and set as docking grids in PyRx. The ligand structures were imported in appropriate formats (e.g., SDF or PDB), followed by energy minimization within PyRx using the built-in universal force field (UFF) algorithm to ensure stable conformations. Ligands were prepared to allow rotatable bonds for flexibility during docking. Docking simulations were performed with the AutoDock Vina engine integrated in PyRx version 0.8. The receptor structures were kept rigid, while the ligands were allowed full flexibility. Multiple docking poses were generated for each ligand, and the best-ranked binding modes were selected based on the lowest binding energy scores (kcal/mol). Docking results, including binding affinities and poses, were analyzed and visualized using PyRx and further inspected with Discovery Studio Visualizer 2019 to assess key interactions between ligands and amino acid residues within the active site. To validate the docking protocol, the co-crystallized ligands from each protein structure were re-docked into their respective binding sites, and the root mean square deviation (RMSD) of their predicted poses relative to the crystallographic positions was calculated. An RMSD value within 2.0 Å confirmed the reliability and reproducibility of the docking procedure.

### 2.12. Statistical Analysis

The statistical analysis was performed using GraphPad Prism 10.5 software. Data were expressed as mean ± SD. Analysis of variance (ANOVA) tests followed by Tukey’s post hoc test were used for comparison. A *p*-value of less than 0.05 was considered statistically significant for all analyses.

## 3. Results

### 3.1. Qualitative Phytochemical Analysis

The preliminary phytochemical screening of the methanolic extract of *J. integerrima* revealed the presence of various bioactive constituents. The alkaloid tests (Mayer’s, Dragendorff’s, Wagner’s, and Hager’s) showed positive results, indicating that alkaloids are a major component of the extract, which may contribute to its potential antimicrobial and pharmacological activities [31]. Flavonoids were also detected by Shinoda, lead acetate, and alkaline reagent tests, suggesting the extract may possess antioxidant and anti-inflammatory properties [32,33]. Similarly, phenolic compounds were confirmed by lead acetate and ferric chloride tests, further supporting the antioxidant potential [34]. The Salkowski test indicated the presence of terpenoids, compounds often associated with antimicrobial, anticancer, and anti-inflammatory activities [35,36]. The foam test confirmed the presence of saponins, which are known for their surfactant, immunomodulatory, and cholesterol-lowering effects [37].

### 3.2. Quantitative Screening

The total flavonoid content of the methanolic extract of *J. integerrima* was determined using the aluminum chloride colorimetric assay with quercetin as the standard (R^2^ = 0.9842). The mean absorbance of the MEJI sample (40 ppm) was 0.313, corresponding to 21.38 µg/mL. The TFC was calculated as 53.45 mg QE/g of extract. The total phenolic content of the methanolic extract of *Jatropha integerrima* was determined using the Folin–Ciocalteu method with gallic acid as the standard (R^2^ = 0.9908). The mean absorbance of the MEJI sample (40 ppm) was 0.126, corresponding to 6.322 mg GAE/mL. The TPC was calculated as 63.22 mg GAE/g of extract.

### 3.3. In Vitro Radical Scavenging Assay

The antioxidant activity of the methanolic extract of *J. integerrima* was evaluated by assessing its ability to reduce DPPH, a stable free radical. The interaction between the antioxidants and DPPH involves the transfer of a hydrogen atom or electron to the DPPH radical, leading to its conversion to 1,1-diphenyl-2-picrylhydrazine [38]. The MEJI demonstrated a concentration-dependent free radical scavenging activity, which is presented in Table 1, ranging from 16.26% to 72.46%. The IC_50_ values for the methanolic extract were calculated at 33.72 µg/mL, while the standard displayed an IC_50_ value of 28.72 µg/mL. Data are expressed as mean ± SD (n = 3). Statistical comparison between ascorbic acid and MEJI at each concentration was performed using a paired *t*-test; *p* < 0.01 was considered statistically significant.

### 3.4. Acute Toxicity Study

An acute oral toxicity study of the methanolic extract of *Jatropha integerrima* was conducted according to the Organization of Economic Co-operation and Development (OECD) Guideline No. 425. No signs of toxicity were observed. No changes in skin and fur, eyes and mucous membranes, or behavior pattern were observed. The safe dose was found to be 5000 mg/kg.

### 3.5. Effect of J. integerrima Extract on Biological Parameters

#### 3.5.1. Effect of MEJI on Body Weight

**Figure 1:**
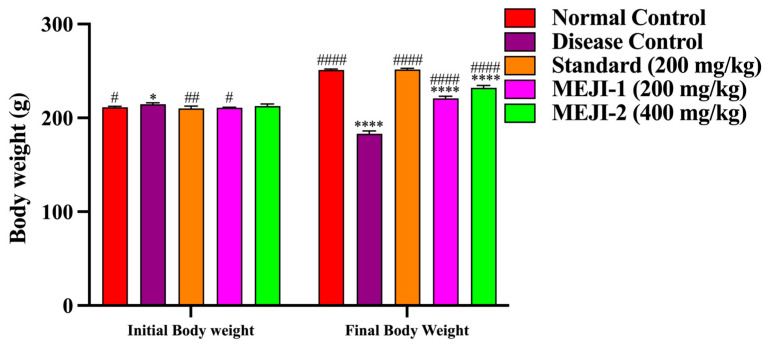
The effects of treatments such as standard and methanolic extract of *Jatropha integerrima* on the body weight of overnight-fasted streptozotocin-induced diabetes (STZ; 45 mg/kg of body weight) in Wistar albino rats. Values are expressed as mean ± standard deviation (n = 5). Two-way ANOVA. * *p* < 0.05, **** *p* < 0.0001 versus normal control group, and ^#^ *p* < 0.05, ^##^ *p* < 0.01, ^####^ *p* < 0.0001 versus diabetes control. NC: normal control; DC: disease control; STD: metformin (200 mg/kg); MEJI 200: methanolic extract of *Jatropha integerrima* (200 mg/kg); MEJI 400: methanolic extract of *Jatropha integerrima* (400 mg/kg).

The results for changes in body weight across experimental groups are presented in Figure 1. At baseline, no significant differences were observed among groups; however, on day 21, the normal control showed an 18.9% gain, whereas the diabetic control exhibited a 14.3% loss (*p* < 0.001 vs. normal), while the metformin-treated rats gained 21.0% (*p* < 0.001 vs. DC), restoring normal levels. MEJI increased body weight by 4.6% at MEJI 200 mg/kg (*p* < 0.05 vs. DC) and 10.9% at MEJI 400 mg/kg (*p* < 0.01 vs. DC), indicating dose-dependent protection against diabetes-induced weight loss, though less effective than metformin.

#### 3.5.2. Effect of MEJI on Fasting Blood Glucose Level

The results of the FBG level, as presented in Table 2, demonstrate that at day 0, FBG levels were comparable across groups (≈ 85–95 mg/dL). However, on day 21, the diabetic control showed a 280.9% rise in FBG (*p* < 0.001 vs. normal), whereas metformin reduced glucose level by 63.4% (*p* < 0.001 vs. DC), restoring it to near-normal levels. MEJI lowered FBG by 48.1% at MEJI 200 mg/kg (*p* < 0.01 vs. DC) and 55.2% at MEJI 400 mg/kg (*p* < 0.001 vs. DC), demonstrating significant and dose-dependent antihyperglycemic activity, with the higher dose of MEJI 400 mg/kg nearly approaching the effect of the standard.

#### 3.5.3. Effect of MEJI on Oral Glucose Tolerance Test

**Figure 2:**
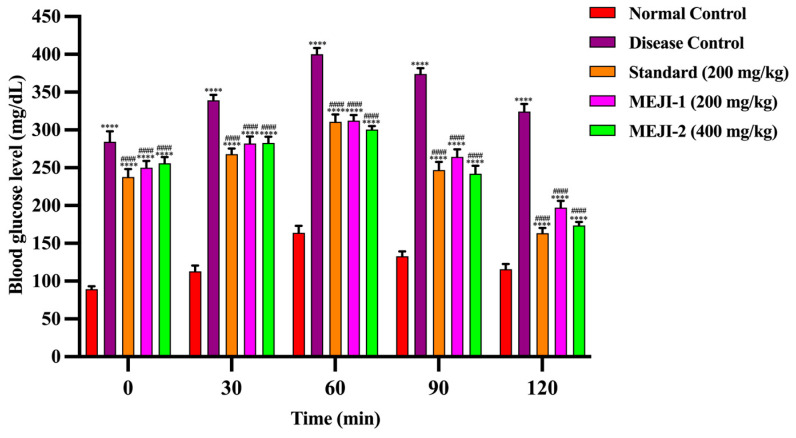
The effects of treatments such as standard and methanolic extract of *Jatropha integerrima* over streptozotocin-induced diabetes (STZ; 45 mg/kg of body weight) in Wistar albino rats on blood glucose level (mg/dL). Values are expressed as mean ± standard deviation (n = 5). Two-way ANOVA with post hoc Tukey’s test. **** *p* < 0.0001 versus normal control group, and #### *p* < 0.0001 versus disease control. NC: normal control; DC: disease control; STD: metformin (200 mg/kg); MEJI 200: methanolic extract of *Jatropha integerrima* (200 mg/kg); MEJI 400: methanolic extract of *Jatropha integerrima* (400 mg/kg).

The OGTT results showed a marked rise in glucose levels in the diabetic control group, peaking at 404.4 ± 0.81 mg/dL at 60 min and remaining elevated, confirming impaired tolerance. The normal control group displayed a modest increase, with values returning near baseline at 120 min, whereas the standard drug significantly improved glucose handling, reducing levels to 140.8 ± 0.58 mg/dL by 120 min. The MEJI treatment demonstrated a dose-dependent effect as the MEJI 200 mg/kg group declined to 191.8 ± 0.66 mg/dL, and the 400 mg/kg group declined to 174.2 ± 0.73 mg/dL at 120 min. Although normal levels were not restored, MEJI 400 mg/kg markedly attenuated hyperglycemia and approached the efficacy of the standard drug (Figure 2).

### 3.6. Effect of J. integerrima Extract on Biological Parameters

#### 3.6.1. Effect of MEJI on Body Weight

**Figure 3:**
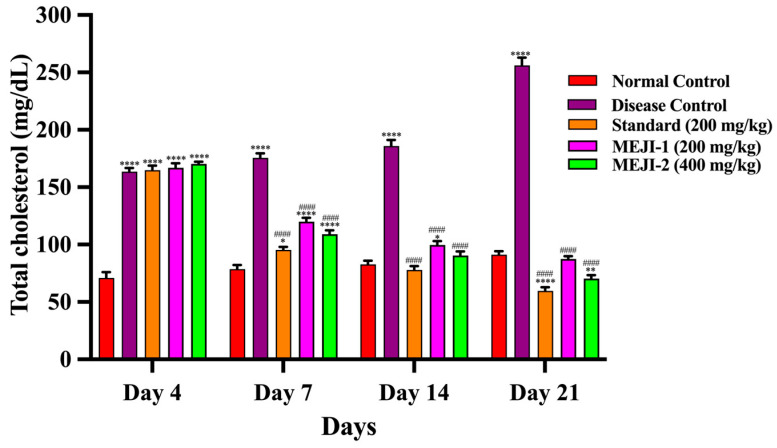
The effects of treatments such as standard and methanolic extract of *Jatropha integerrima* over streptozotocin-induced diabetes (STZ; 45 mg/kg of body weight) in Wistar albino rats on total cholesterol. Values are expressed as mean ± standard deviation (n = 5). Two-way ANOVA with post hoc Tukey’s test. * *p* < 0.05, ** *p* < 0.01, **** *p* < 0.0001 versus normal control group, and #### *p* < 0.0001 versus diabetic control. NC: normal control; DC: disease control; STD: metformin (200 mg/kg); MEJI 200: methanolic extract of *Jatropha integerrima* (200 mg/kg); MEJI 400: methanolic extract of *Jatropha integerrima* (400 mg/kg).

At baseline, TC levels were comparable across groups. By day 21, the diabetic control group exhibited a marked 279% increase (*p* < 0.001 vs. normal), confirming diabetes-induced hypercholesterolemia, whereas the standard treatment reduced TC by 72% (*p* < 0.001 vs. DC), restoring near-normal levels. MEJI 200 mg/kg decreased TC by 62% (*p* < 0.01 vs. DC), while MEJI 400 mg/kg showed a stronger effect with a 66% reduction (*p* < 0.001 vs. DC), demonstrating dose-dependent antihyperlipidemic activity comparable to that of the standard drug (Figure 3).

#### 3.6.2. Effect of MEJI on TG Level

**Figure 4:**
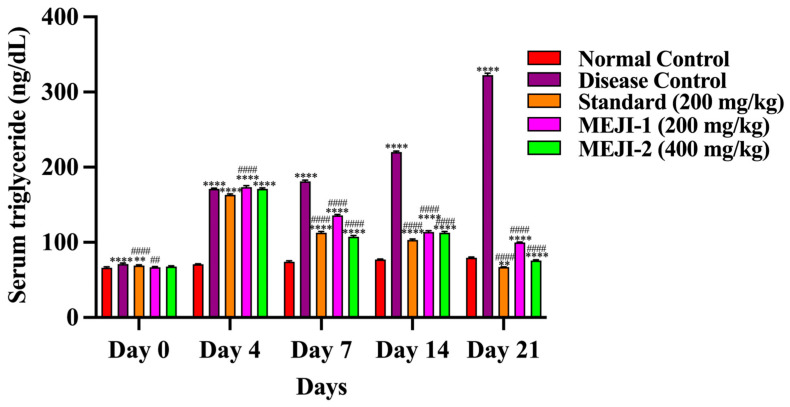
The effects of treatments such as standard and methanolic extract of *Jatropha integerrima* over streptozotocin-induced diabetes (STZ; 45 mg/kg of body weight) in Wistar albino rats on total triglyceride (TG). Values are expressed as mean ± SD (n = 5). One-way ANOVA with post hoc Tukey’s test. ** *p* < 0.01, **** *p* < 0.0001 versus normal control group, and ## *p* < 0.01, #### *p* < 0.0001 versus diabetic control. NC: normal control; DC: disease control; STD: metformin (200 mg/kg); MEJI 200: methanolic extract of *Jatropha integerrima* (200 mg/kg); MEJI 400: methanolic extract of *Jatropha integerrima* (400 mg/kg).

TG levels were similar across groups, as presented in Figure 4. By day 21, the diabetic control group showed a 353% rise (*p* < 0.001 vs. normal), confirming diabetes-induced hypertriglyceridemia, while the standard treatment reduced TG by 79% (*p* < 0.001 vs. DC), restoring near-normal levels. However, the treatment of animals with MEJI at 200 mg/kg lowered TG by 69% (*p* < 0.05 vs. DC), whereas MEJI 400 mg/kg achieved a stronger 77% reduction (*p* < 0.01 vs. DC), indicating a dose-dependent antihyperlipidemic effect comparable to the standard drug.

#### 3.6.3. Effect of MEJI on HDL Level

**Figure 5:**
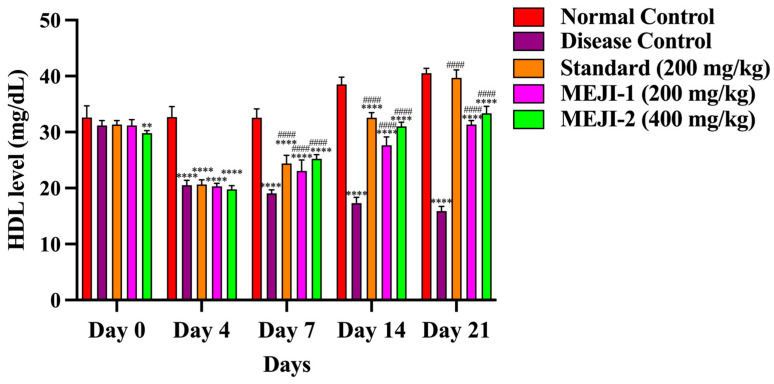
The effects of treatments such as standard and methanolic extract of *Jatropha integerrima* over streptozotocin-induced diabetes (STZ; 45 mg/kg of body weight) in Wistar albino rats on HDL. Values are expressed as mean ± SD (n = 5). One-way ANOVA with post hoc Tukey’s test. ** *p* < 0.01, **** *p* < 0.0001 versus normal control group, and #### *p* < 0.0001 versus diabetic control. NC: normal control; DC: disease control; STD: metformin (200 mg/kg); MEJI 200: methanolic extract of *Jatropha integerrima* (200 mg/kg); MEJI 400: methanolic extract of *Jatropha integerrima* (400 mg/kg).

HDL levels were comparable across groups. By day 21, the diabetic control group showed a 59% reduction (*p* < 0.001 vs. normal), confirming diabetes-induced loss of protective HDL. Standard treatment increased HDL by 141% (*p* < 0.001 vs. DC), restoring near-normal levels. MEJI at 200 mg/kg improved HDL by 93% (*p* < 0.05 vs. DC), while 400 mg/kg produced a stronger 105% rise (*p* < 0.01 vs. DC). MEJI 400 displayed HDL-raising potential comparable to the standard, highlighting MEJI’s protective role in ameliorating diabetic dyslipidemia (Figure 5).

#### 3.6.4. Effect of MEJI on LDL Level

**Figure 6:**
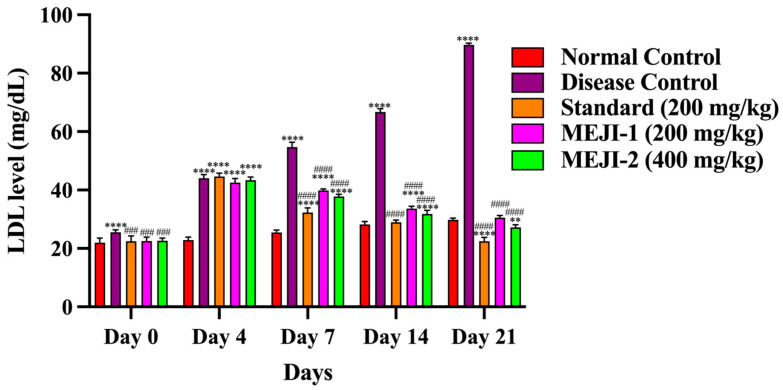
The effects of treatments such as standard and methanolic extract of *Jatropha integerrima* over streptozotocin-induced diabetes (STZ; 45 mg/kg of body weight) in Wistar albino rats on LDL. Values are expressed as mean ± SD (n = 5). One-way ANOVA with post hoc Tukey’s test. ** *p* < 0.01, **** *p* < 0.0001 versus normal control group, and ### *p* < 0.001, #### *p* < 0.0001 versus diabetic control. NC: normal control; DC: disease control; STD: metformin (200 mg/kg); MEJI 200: methanolic extract of *Jatropha integerrima* (200 mg/kg); MEJI 400: methanolic extract of *Jatropha integerrima* (400 mg/kg).

LDL levels showed a marked elevation in the diabetic control group by day 21, with a 238% rise (*p* < 0.001 vs. normal), confirming diabetes-induced increase in LDL. Standard (metformin) treatment reduced LDL by 74% (*p* < 0.001 vs. DC), restoring it to near normal. MEJI 200 mg/kg decreased LDL by 65% (*p* < 0.05 vs. DC), while 400 mg/kg achieved a stronger 68% reduction (*p* < 0.01 vs. DC), showing dose-dependent antihyperlipidemic efficacy comparable to the standard, further indicating strong antihyperlipidemic activity (Figure 6).

### 3.7. Effect of MEJI on SOD and CAT Levels

Diabetic animals exhibited a marked 66% decline in SOD activity (*p* < 0.001 vs. normal), reflecting impaired antioxidant defense. Treatment with the standard drug markedly restored SOD, showing a 156% elevation (*p* < 0.001 vs. DC). MEJI supplementation also improved enzyme levels, with the MEJI 200 mg/kg dose raising SOD by 76% (*p* < 0.05 vs. DC), and the MEJI 400 mg/kg dose producing a more pronounced 111% increase (*p* < 0.01 vs. DC), highlighting a clear dose-dependent trend. CAT activity was substantially reduced in the diabetic control group (58% lower than normal, *p* < 0.001). Administration of the standard treatment normalized enzyme function, yielding a 119% increase (*p* < 0.001 vs. DC). MEJI produced notable improvements as well. MEJI 200 mg/kg enhanced catalase by 77% (*p* < 0.05 vs. DC), while MEJI 400 mg/kg boosted it by 101% (*p* < 0.01 vs. DC), demonstrating effective restoration of antioxidant capacity in a dose-dependent manner Table 3.

### 3.8. Histopathological Analysis

**Figure 7:**
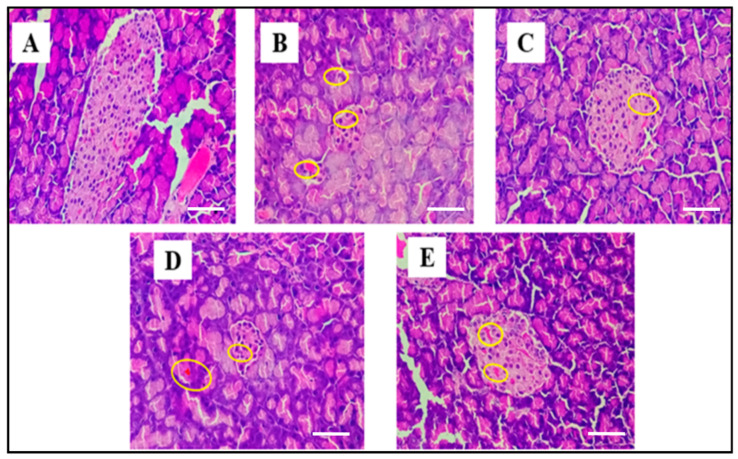
Histopathological analysis of pancreatic cells after 21 days of treatment with test and standard (400×; scale bars = 25 µm). Normal control (**A**); diabetic control (**B**); standard: metformin (200 mg/kg) (**C**); MEJI 200: methanolic extract of *Jatropha integerrima* (200 mg/kg) (**D**); MEJI 400: methanolic extract of *Jatropha integerrima* (400 mg/kg) (**E**).

Histopathological analysis showed normal islet architecture in the control group (A), while the diabetic control group exhibited severe β-cell degeneration with shrunken and vacuolated islets (B). Standard (metformin 200 mg/kg) treatment restored islet structure significantly (C). MEJI 200 mg/kg showed moderate recovery, whereas MEJI 400 mg/kg demonstrated marked regeneration with preserved islet morphology, indicating dose-dependent pancreatic protection (D and E) (Figure 7).

### 3.9. Molecular Docking

**Figure 8:**
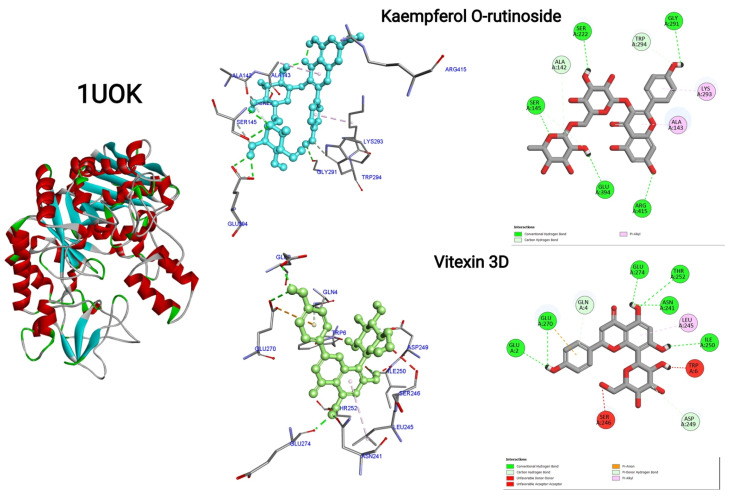
Two-dimensional and three-dimensional interactions of Kaempferol O-rutinoside and Vitexin D for PDB ID: 1UOK.

**Figure 9:**
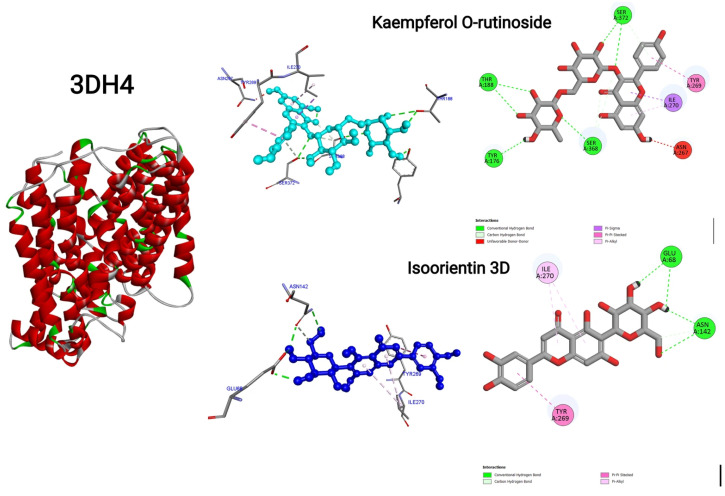
Two-dimensional and three-dimensional interactions of Kaempferol O-rutinoside and Isoorientin 3D for PDB ID: 3DH4.

**Figure 10:**
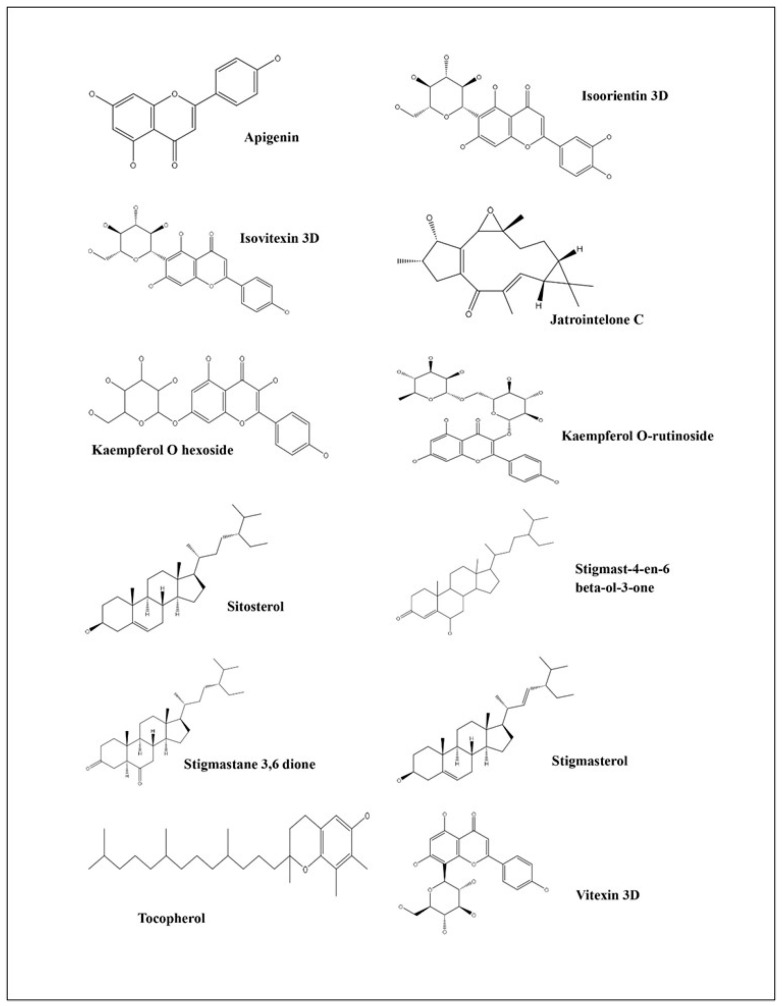
Chemical structures of major phytochemicals present in *Jatropha integerrima*.

The molecular docking evaluation of twelve phytochemicals against the antidiabetic target proteins 1UOK and 3DH4 reveals strong ligand–receptor complementarity, suggesting the potential of the plant extract as a natural source of antidiabetic agents. Across both proteins, flavonoids and flavonoid glycosides exhibited the lowest binding energies and the highest frequency of stabilizing interactions, indicating their strong inhibitory potential toward key enzymes involved in glucose metabolism. Among all screened molecules, Kaempferol O-rutinoside demonstrated the strongest binding (−9.4 kcal/moL for 1UOK, −9.8 kcal/moL for 3DH4), forming an extensive network of hydrogen bonds with residues such as SER145, SER222, GLY291, GLU394, TYR176, THR188, SER368, and SER372. These interactions are essential for stabilizing ligand occupancy in the catalytic pocket, strongly supporting its role as a potent inhibitor. Similarly, Kaempferol O-hexoside, Isoorientin, Isovitexin, and Vitexin showed high affinity (−8.3 to −9.2 kcal/moL), engaging in multiple conventional hydrogen bonds, π–π stacking (PHE163, TYR263, TYR269), π-donor interactions, π-anion contacts, and carbon–hydrogen bonds, all of which enhance inhibitory performance against both proteins. The basic flavonoid Apigenin also displayed strong binding (−8.0 to −8.2 kcal/moL), driven by several π–π and H-bond interactions with catalytic residues such as TYR263, PHE163, and ASP329, indicating its capability to modulate receptor activity. These flavonoid–protein interactions are consistent with previously reported glucose-lowering mechanisms, including α-glucosidase suppression, PTP1B inhibition, and improved insulin sensitization. Phytosterols such as Sitosterol, Stigmasterol, and Stigmastane derivatives exhibited moderate binding (−6.8 to −9.0 kcal/moL) dominated by hydrophobic contacts (π-alkyl and alkyl interactions) with residues like ALA143, PHE163, VAL200, and LEU443. Their preference for hydrophobic pockets reflects their known biological roles in membrane stabilization and insulin signaling enhancement. Although their binding energies were slightly lower than those of flavonoids, the strong hydrophobic stabilization suggests possible synergistic antidiabetic effects. Tocopherol, while showing comparatively lower affinity (−6.6 to −7.2 kcal/moL), maintained significant π-alkyl and hydrogen bonding contacts with GLU255, PHE172, LYS293, and TYR176. This supports its role as an auxiliary antioxidant contributing to overall metabolic regulation. Conclusively, the docking results consistently indicate that flavonoids and flavonoid glycosides possess superior binding affinity and structural complementarity toward both antidiabetic enzymes. Their ability to interact through multiple hydrogen bonds, aromatic stacking, and anion–π contacts gives them a biochemical advantage in inhibiting protein activity associated with hyperglycemia. In contrast, sterols and tocopherols provide additional hydrophobic contributions that may complement the action of flavonoids in the plant extract (Figure 8, Figure 9 and Figure 10). Thus, the combined molecular evidence strongly supports the antidiabetic potential of the plant extract, highlighting flavonoid glycosides such as Kaempferol O-rutinoside, Isoorientin, Isovitexin, Kaempferol O-hexoside, and Vitexin as the most promising bioactive constituents. These findings justify further in vitro enzymatic inhibition studies and in vivo antidiabetic assays to validate their therapeutic relevance Table 4.

## 4. Discussion

Diabetes *mellitus* is a metabolic disease marked by higher blood glucose levels driven by either insulin malfunction or insufficiency [39]. With an alarming rate of growth in recent years, diabetes is one of the world’s leading causes of human suffering and mortality. Diabetes is growing more prevalent, which emphasizes how urgently novel methods of treatment must be explored [40]. Despite the availability of numerous glucose-lowering medications, their effectiveness has been limited by resistance, side effects, and cytotoxicity. Plant-based medicines are becoming increasingly renowned due to their benefits, as they are safe to use and non-hazardous [41]. The previous studies reported that the genus *Jatropha gossypifolia* Linn and *Jatropha curcas* show antioxidant, antidiabetic, and anti-glycemic properties. The studies demonstrated that *Jatropha integerrima* extract may show antidiabetic potential in animal models at lower dose levels [42,43,44].

The present study was undertaken to evaluate the antidiabetic, antihyperlipidemic, and antioxidant potential of the methanolic extract of *Jatropha integerrima* leaves in STZ-induced type 2 diabetic rat models. STZ is the most commonly used drug to induce diabetes in experimental animals. STZ is transported to the pancreatic beta cells by the plasma membrane’s GLUT-2 transporter. STZ builds up in β-cells, and DNA alkylating agents cause cell necrosis, which lowers insulin production and its release and triggers hyperglycemia [45]. The STZ-induced diabetic model is a well-established method to mimic T2DM, characterized by the selective destruction of pancreatic β-cells, leading to insulin deficiency and persistent hyperglycemia [46]. This model also allows for the investigation of secondary complications such as dyslipidemia, oxidative stress, and weight loss, all of which are hallmarks of uncontrolled diabetes [47].

The MEJI exhibited a diverse phytochemical profile comprising alkaloids, flavonoids, phenolics, terpenoids, and saponins, reflecting its broad biological potential. The presence of flavonoids and phenolic compounds is closely associated with the observed antioxidant activity, as these constituents are known to effectively scavenge free radicals through hydrogen or electron donation. The radical scavenging ability demonstrated in the DPPH assay supports the role of these phenolic and flavonoid compounds as primary contributors to the antioxidant effect. In addition, terpenoids and saponins may exert complementary effects by enhancing overall bioactivity through synergistic interactions. Collectively, the findings indicate that the antioxidant potential of *J. Integerrima* is intrinsically linked to its rich and complex phytochemical composition. In the current study, STZ administration significantly elevated blood glucose levels in diabetic rats, confirming the successful induction of diabetes. The methanolic extract of *Jatropha integerrima*, administered in two different doses, exhibited a significant and dose-dependent reduction in fasting blood glucose levels compared to the diabetic control group. This hypoglycemic effect was comparable, though slightly less potent, than the standard drug (metformin), suggesting that the extract demonstrated significant glucose-lowering activity compared to the diabetic control and that further mechanistic studies are required to elucidate the underlying pathways [48]. The mechanism could be attributed to the presence of phytoconstituents such as flavonoids, phenols, and saponins, which have previously been reported to stimulate glucose uptake, promote insulin sensitivity, or regenerate damaged β-cells [49].

Body weight loss is another clinical manifestation of diabetes *mellitus*. This is due to poor glycemic control, which promotes tissue wasting that accelerates the metabolism of fat and protein in diabetic rats [1]. The STZ-induced diabetes rats had significantly lost weight, primarily as a result of their muscles losing more glucose than they used as energy [50]. Administration of MEJI or metformin to diabetic rats resulted in a substantial increase in body weight. By promoting glucose utilization in insulin target tissues while minimizing the activities of the gluconeogenic enzymes, MEJI or metformin may have an inhibitory effect on glucose metabolism, minimizing muscle wasting.

Persistent hyperglycemia, along with impaired glucose tolerance and increased gluconeogenesis, are some of the characteristic features of diabetes *mellitus* [51]. In this study, constant hyperglycemia and impaired glucose tolerance were observed in diabetic rats [52]. Treatment of diabetic rats with the 200 and 400 mg/kg MEJI or metformin significantly reduced the blood sugar concentrations, with improvement in the oral glucose tolerance test and enhanced insulin sensitivity. The fasting blood glucose levels of 400 mg/kg MEJI and metformin are lower compared to 200 mg/kg MEJI-treated diabetic rats, suggesting the good antidiabetic potential of the 400 mg/kg MEJI dose. The data corroborated the findings of histopathological studies, where the MEJI 400 mg/kg with treatment to diabetic rats resulted in a significant increase in islet area and insulin-positive cells as compared to MEJI 200 mg/kg treated diabetic rats. These results indicate the shielding effect of MEJI on the β-cells of the pancreas through its anti-diabetic potential and thereby restore the normal functioning of cells. The improvement in these metabolic alterations proved the effectiveness of *J. integerrima* leaves extract in combating diabetic complications through its antidiabetic nature.

One prominent complication of T2DM is hyperlipidemia, which may be a risk factor for cardiovascular disease [53]. Insulin stimulates lipoprotein lipase, which breaks down triglycerides into fatty acids and glycerol under normal physiological conditions. After that, these fatty acids are either oxidized to provide energy or re-esterified to store it in the tissues of the body. Insulin resistance and/or deficiency in diabetics causes lipoprotein lipase to become inactive, resulting in hyperglycemia [54]. While HDL carries endogenous cholesterol from body tissues to the liver for metabolism and elimination, a normal biological level of LDL influences the movement of cholesterol from the liver to other body tissues. While high levels of HDL prevent atherosclerosis by preventing cholesterol deposition, elevated levels of LDL trigger coronary heart disease by accumulating cholesterol in the arteries [55]. In this study, the induced type 2 diabetic condition in rats resulted in an increased level of total cholesterol, triglycerides, and LDL, in association with reduced levels of HDL. The administration of 200 and 400 mg/kg *J. integerrima* leaves extract or metformin to diabetic rats markedly improved the hyperlipidemic condition in rats by either improved secretion of insulin/or enhanced insulin sensitivity. Insulin is a potent inhibitor of lipolysis, and therefore, during diabetes *mellitus*, the activity of the lipase enzyme increases lipolysis and releases more free fatty acids in the circulation because of the lack of insulin [56,57]. An increase in fatty acid concentration in turn increases the beta-oxidation of fatty acids by increasing the activity of β-Hydroxyl-β-methylglutaryl Coenzyme-A (HMG-CoA) reductase for the production of more cholesterol [58]. Moreover, insulin facilitates the receptor-mediated clearance of low-density lipoprotein cholesterol (LDL cholesterol), and hypercholesterolemia is brought on by lower insulin activity in diabetes *mellitus* [59]. These abnormalities were lessened in the groups that received MEJI extract therapy; this could be attributed to the fact that insulin secretion improved as hyperglycemia decreased, which in turn hindered lipolysis. Additionally, due to their antioxidant abilities, phytochemicals like saponins have been proven to obstruct the oxidation of fatty acids [60]. Thus, indicating that the *J. integerrima* leaves extract possesses hypolipidemic properties, and this may help limit the incidence of cardiovascular diseases like atherosclerosis associated with diabetes *mellitus*.

Reactive oxygen species-induced oxidative stress is believed to be a prevalent mechanism in difficulties associated with diabetes. When oxidants and antioxidant defenses are out of balance, oxidative stress occurs in cells [61]. In this study, the markers of oxidative stress, such as SOD and CAT levels, were significantly decreased in the pancreas of diabetic rats. While the administration of 200 or 400 mg/kg of MEJI to diabetic rats caused a significant improvement in markers of oxidative stress, suggesting the antioxidant effect of MEJI on diabetes-induced oxidative stress. The treatment of MEJI 400 in diabetic rats significantly improved the metabolic alterations, fasting blood glucose level, dyslipidemia, and oxidative stress through its antidiabetic, antihyperlipidemic, and anti-oxidative effects. Therefore, the methanolic extract of *J. integerrima* could be an ideal treatment option for diabetes *mellitus.*

## 5. Conclusions

The present study demonstrated that the methanolic extract of *Jatropha integerrima* possesses significant antidiabetic, antihyperlipidemic, and antioxidant effects in streptozotocin-induced diabetic rats. MEJI not only reduced fasting blood glucose and improved glucose tolerance but also favorably modulated the lipid profile and enhanced antioxidant enzyme levels, thereby mitigating oxidative stress. Histopathological findings further revealed protective and regenerative effects on pancreatic β-cells. These results suggest that *Jatropha integerrima* holds promise as a potential phytotherapeutic agent for the management of diabetes and its associated complications.

## Figures and Tables

**Table 1:** Percentage inhibition by DPPH radical scavenging assay.

Concentration (µg/mL)	Percentage Inhibition of DPPH	
	Ascorbic Acid (Standard)	MEJI
10	26.57 ± 0.77	16.26 ± 0.21 **
20	39.89 ± 0.69	29.09 ± 0.42 **
30	57.84 ± 0.78	48.42 ± 0.42 **
40	73.68 ± 0.37	62.15 ± 0.43 **
50	86.27 ± 0.63	72.46 ± 0.56 **
IC_50_ value	28.72 µg/mL	33.72 µg/mL

Values are in mean ± SD, (n = 3); paired-*t* test with confidence interval of 99.0%; ** *p* < 0.01.

**Table 2:** The effects of treatments such as standard and methanolic extract of *Jatropha integerrima* on the fasting blood glucose level of the tested animals, as measured using a glucometer.

Days	Treatment Groups				
	Normal	Diabetic Control	Standard	MEJI 200	MEJI 400
Day 0	94.8 ± 0.73	90.2 ± 0.37	92.4 ± 0.81	84.6 ± 0.95	93 ± 0.89
Day 4	97.8 ± 0.8	266.8 ± 0.58 ^###^	260.8 ± 0.58	256.8 ± 0.94	266.2 ± 0.86
Day 7	100.8 ± 0.6	309.4 ± 0.87 ^###^	174.2 ± 0.86 ***	201.8 ± 0.74 *	181.6 ± 0.74 **
Day 14	103 ± 0.7	323 ± 0.89 ^###^	135 ± 0.83 ***	187.4 ± 0.32 *	154.6 ± 0.87 **
Day 21	94.2 ± 0.8	361.4 ± 0.74 ^###^	95.54 ± 0.78 ***	133.2 ± 0.94 *	119.2 ± 0.58 **

Values are expressed as mean ± standard deviation (n = 5). Two-way ANOVA with post hoc Tukey’s test. * *p* < 0.05, ** *p* < 0.01, *** *p* < 0.001 versus diabetic control group, and ### *p* < 0.001 versus normal control. Standard: metformin (200 mg/kg); MEJI 200: methanolic extract of *Jatropha integerrima* (200 mg/kg); MEJI 400: methanolic extract of *Jatropha integerrima* (400 mg/kg).

**Table 3:** The effects of treatments such as standard and methanolic extract of *Jatropha integerrima* in the tissue homogenate of liver on SOD and CAT levels.

Antioxidant Enzymes	Treatment Groups				
	Normal	Diabetic Control	Standard	MEJI 200	MEJI 400
SOD ±SD	11.13 ± 0.19	3.83 ± 0.21 ^###^	9.79 ± 0.13 ***	6.74 ± 0.08 *	8.08 ± 0.08 **
CAT ±SD	8.13 ± 0.08	3.4 ± 0.13 ^###^	7.45 ± 0.12 ***	6.01 ± 0.06 *	6.83 ± 0.09 **

Values are expressed as mean ± SD (n = 5). Two-way ANOVA with post hoc Tukey’s test. * *p* < 0.05, ** *p* < 0.01, *** *p* < 0.001 versus diabetic control group, and ### *p* < 0.001 versus normal control. Standard: metformin (200 mg/kg); MEJI 200: methanolic extract of *Jatropha integerrima* (200 mg/kg); MEJI 400: methanolic extract of *Jatropha integerrima* (400 mg/kg).

**Table 4:** Binding affinities and protein interactions of selected phytochemicals.

Phytochemical	Protein Binding Affinity IDs		Type of Bonding and Interaction
	1UOK	3DH4	
Apigenin	−8	−8.2	Pi-Alkyl/Alkyl—VAL A:200, PRO A:257 Pi-Pi Stacked—PHE A:163, PHE A:281 Pi-Pi T-shaped—PHE A:163, PHE A:281 Pi-Anion—ASP A:329, GLU A:255
			Conventional H-bond—TYR A:263 Pi-Sigma—ILE A:270 Pi-Alkyl/Alkyl—ILE A:270 Pi-Pi Stacked—TYR A:263, TYR A:269 Pi-Pi T-shaped—TYR A:263, TYR A:269 Pi-Donor H-bond—ASN A:26, SER A:372
Isoorientin 3D	−8.3	−9.1	Conventional H-bond—TYR A:220, SER A:222, ASP A:329 Pi-Sigma—ALA A:143 Pi-Alkyl/Alkyl—PRO A:257 Carbon H-bond—ALA A:142 Pi-Pi T-shaped—PHE A:163
			Conventional H-bond—ARG A:380, ASP A:394, ASN A:420, ARG A:423 Pi-Anion—ASP A:373, ARG A:380, ASP A:421 Pi-Cation—ASP A:373, ARG A:380, ASP A:421
Isovitexin 3D	−8.4	−8.6	Conventional H-bond—SER A:145, GLY A:291, ASP A:329, GLU A:394 Pi-Sigma—ALA A:143 Pi-Alkyl/Alkyl—ALA A:143 Carbon H-bond—TRP A:294 Pi-Pi T-shaped—PHE A:163
			Conventional H-bond—GLU A:68, ASN A:142, TYR A:262, ASN A:267 Pi-Alkyl/Alkyl—ILE A:270 Carbon H-bond—TYR A:138, GLN A:268, SER A:372 Pi-Pi T-shaped—TYR A:269 Pi-Donor H-bond—TYR A:138, GLN A:268, SER A:372
Jatrointelone C	−7.9	−8.5	Pi-Alkyl/Alkyl—ALA A:143, PHE A:227, MET A:228, PRO A:257
			Conventional H-bond—PHE A:133
Kaempferol O hexoside	−8.8	−9.1	Conventional H-bond—SER A:145, ASP A:329, GLU A:387, GLU A:394 Pi-Sigma—ALA A:143 Pi-Alkyl/Alkyl—ALA A:143
			Conventional H-bond—SER A:66, GLN A:69, SER A:365, SER A:372 Pi-Sigma—MET A:369 Pi-Alkyl/Alkyl—MET A:369 Carbon H-Bond—TYR A:263, SER A:368, SER A:372 Pi-Pi T-shaped—TYR A:263 Pi-Donor H-bond—TYR A:263, SER A:368, SER A:372 Pi-Anion—GLU A:68
Kaempferol O rutinoside	−9.4	−9.8	Conventional H-bond—SER A:145, SER A:222, GLY A:291, GLU A:394, ARG A:415 Pi-Alkyl/Alkyl—ALA A:143, LYS A:293 Carbon H-bond—ALA A:142, SER A:145, TRP A:294
			Conventional H-bond—TYR A:176, THR A:188, SER A:368, SER A:372 Pi-Sigma—ILE A:270 Pi-Alkyl/Alkyl—ILE A:270 Carbon H-bond—SER A:368, SER A:372 Pi-Pi Stacked—TYR A:269 Pi-Donor H-bond—SER A:368, SER A:372
Sitosterol	−8.4	−8.4	Pi-Alkyl/Alkyl—ALA A:143, PHE A:163, VAL A:200
			Pi-Sigma—TYR A:430 Pi-Alkyl/Alkyl—VAL A:434
Stigmastane-4-en-6 beta-ol-3-one	−6.8	−8.4	Pi-Alkyl/Alkyl—ALA A:143, PHE A:203, PRO A:257, PHE A:281
			Conventional H-bond—TYR A:426 Pi-Alkyl/Alkyl—LEU A:433, VAL A:434, ILE A:464
Stigmastane3,6 dione	-	−8.7	-
			Pi-Alkyl/Alkyl—VAL A:434
Stigmasterol	−8	−9	Pi-Alkyl/Alkyl—VAL A:296, ARG A:336, ILE A:412 Carbon H-bond—PRO A:298
			Pi-Alkyl/Alkyl—LEU A:261, LEU A:443, PHE A:447, LEU A:487
Tocopherol	−6.6	−7.2	Conventional H-bond—GLU A:255 Pi-Alkyl/Alkyl—ALA A:143, PRO A:257, LYS A:293 Pi-Pi T-shaped—HE A:163 25
			Pi-Sigma—PHE A:172, TYR A:176 Pi-Alkyl/Alkyl—PHE A:172, VAL A:175, ALA A:359, VAL A:363 Pi-Pi T-shaped—PHE A:172
Vitexin 3D	−9.2	−8	Conventional H-bond—GLU A:2, ASN A:241, ILE A:250, THR A:252, GLU A:270, GLU A:274 Pi-Alkyl/Alkyl—LEU A:245 Carbon H-bond—GLN A:4, ASP A:249 Pi-Anion—GLU A:270
			Conventional H-bond—TYR A:138, TYR A:263, SER A:368 Pi-Sigma—ILE A:270 Carbon H-bond—ASN A:64, SER A:372 Pi-Pi Stacked—TYR A:269 Pi-Donor H-bond—ASN A:64, SER A:372

## Data Availability

The original contributions presented in the study are included in the article, further inquiries can be directed to the corresponding author.

## Abbreviations

The following abbreviations are used in this manuscript:

DM	Diabetes mellitus
MEJI	Methanolic extract of *Jatropha integerrima*
STZ	Streptozotocin
OGTT	Oral glucose tolerance test
TC	Total cholesterol
TG	Triglyceride
LDL	Low-density lipoprotein
HDL	High-density lipoprotein
VLDL	Very-low-density lipoprotein
SOD	Superoxide dismutase
CAT	Catalase
FBG	Fasting blood glucose
ROS	Reactive oxygen species
DPPH	2,2-diphenyl-1-picrylhydrazyl
IAEC	Institutional Animal Ethics Committee
CCSEA	Committee for the Control and Supervision of Experiments on Animals
OECD	Organization of Economic Co-operation and Development
AUC	Area under the curve
PDB	Protein Data Bank
UFF	Universal force field
RMSD	Root mean square deviation
TFC	Total flavonoid content
TPC	Total phenolic content
GAE	Gallic acid
DC	Disease control
HMG-CoA	β-Hydroxyl-β-methylglutaryl Coenzyme-A
